# Structural innovation under environmental pressure: the evolutionary origins and regulatory mechanisms of thorns

**DOI:** 10.48130/forres-0026-0020

**Published:** 2026-06-18

**Authors:** Zhe Li, Changhui Tian, Dinghai Zhou, Zhiwen Shi, Lingyan Tan, Yongqing Guo, Yanli Hu, Jihua Mao, Xitong Fei

**Affiliations:** 1 College of Forestry/ Shenzhen Research Institute, Northwest Agriculture and Forestry University, Xianyang, Shaanxi 712100/Shenzhen, Guangdong 518000, China; 2 Research Centre for Engineering and Technology of Zanthoxylum State Forestry Administration, Yangling, Xianyang, Shaanxi 712100, China; 3 Key Comprehensive Laboratory of Forestry of Shaanxi Province, Northwest Agriculture and Forestry University, Yangling, Shaanxi 712100, China; 4 Forestry Vocational Technical School of Yunnan Province, Kunming, Yunnan 650201, China

**Keywords:** Thorns, Adaptive evolution, Structure, Function, Regulatory networks, Phytohormone signaling

## Abstract

Plant thorns represent a prominent evolutionary innovation formed through millions of years of natural selection. However, thorn structures greatly restrict the cultivation and management of many economically important crops, making the breeding of thornless varieties a key objective in modern agricultural breeding. Although different plant species possess apparently similar sharp appendages, these structures originate from different plant organs or tissues. Thorns are not merely mechanical defense structures but are the result of evolutionary adaptations by plants to cope with herbivore pressure and abiotic stresses over evolutionary time. Thorn morphogenesis integrates multiple pathways involved in developmental regulation, defense signaling, and environmental perception, reflecting the resource allocation trade-offs between growth investment and defense efficiency in plants. This review provides a comprehensive synthesis of the developmental origins, anatomical diversity, and ecological defense functions of plant thorns. By integrating paleobotanical evidence, this review traces the evolutionary emergence of thorns during the Paleogene and Neogene periods and reveals the adaptive associations between thorn diversification and the radiation of large herbivorous mammals. At the molecular level, this review focuses on recent advances in developmental genetics, with an emphasis on the regulatory networks underlying thorns' development, as well as the regulatory roles and crosstalk mechanisms of phytohormone signaling pathways involved in defense, including jasmonic acid and gibberellin, in thorns' development. Through a multidisciplinary perspective, this review provides a synthesis of current knowledge on the origin, diversification, and environmental adaptive significance of thorns, providing new insights into the developmental plasticity of plant defense structures.

## Introduction

As sessile organisms, plants have evolved diverse defense strategies, among which thorns represent an important structural adaptation in some species^[1]^. Thorns are widely distributed across multiple phylogenetic lineages of angiosperms and gymnosperms. Their repeated independent origins and remarkable convergent evolution reveal a shared adaptive strategy developed by plants to cope with environmental stresses^[2]^. As sessile organisms, plants cannot evade risks through movement; therefore, they have gradually evolved morphological and chemical defenses against herbivory, insect attack, pathogen invasion, and abiotic stresses such as drought and salinity^[3,4]^. Among these, thorns stand out as a key structural innovation within plant defense systems, providing an effective mechanical barrier at a relatively low energy cost.

Thorns have evolved independently multiple times across different lineages, exhibiting typical convergent traits that underscore their universal adaptive advantage in ecosystems^[2]^. According to their developmental origins, these structures can be categorized into three main types: Leaf thorns, stem thorns, and prickles. Leaf thorns are derived from modified leaf organs, stem thorns originate from specialized axillary branches, and prickles develop from epidermal or cortical cells, usually lacking vascular connections and therefore easily detachable^[5]^. These developmental distinctions not only highlight specialization at the organ level but reflect trade-offs between functional efficiency and construction cost that are specific to each species.

Ecologically, thorn formation constitutes a multifaceted adaptive response to both biotic and abiotic pressures. The epidermis of thorns is often highly cutinized or lignified, forming rigid piercing structures that effectively reduce damage from herbivores and insects. In some species, thorns are coupled with chemical defenses; for instance, the stinging hairs of Urticaceae secrete acetic or formic acids, contributing to a synergistic defense mechanism^[6]^. In arid and desert ecosystems, plants such as Cactaceae use dense thorn structures to reduce their transpiration surface area and water loss, thereby maintaining physiological balance under extreme conditions^[7]^. Meanwhile, animals have coevolved adaptive feeding strategies; for example, insect herbivores activate induced defenses both locally and systemically by signaling pathways involving systemin, jasmonate, oligogalacturonic acid, and hydrogen peroxide, thereby illustrating the dynamic equilibrium of thorns within ecological networks^[8]^. Hence, thorns function not merely as physical defense organs but also as crucial nodes that mediate biotic interactions and resource allocation strategies in ecosystems.

Evidence from paleobotany and comparative morphology further indicates that thorns' evolution is closely linked to the radiation of large herbivorous mammals. In African savanna ecosystems, thorn density and length in *Acacia* species are significantly positively correlated with herbivore browsing pressure, whereas in island environments lacking herbivores, the spinescent structures of the same species tend to be reduced or lost^[9]^. This coevolutionary relationship indicates that thorns' morphological characteristics are shaped by both abiotic environmental factors and herbivore selection pressure. Meanwhile, thorn formation is governed by complex genetic and developmental regulatory mechanisms; their elimination or optimization involves the reprogramming of multilayered gene networks and signaling pathways, rather than simple morphological modification^[10,11]^.

In recent years, advances in plant developmental biology and molecular genetics have progressively revealed the molecular basis of thorn initiation, differentiation, and morphogenesis. Transcription factor regulatory networks, phytohormone signaling, and epigenetic modifications have been identified as key factors involved in thorn development, providing new perspectives for understanding the evolutionary logic of plant defense strategies. Elucidating the mechanisms underlying thorn formation deepens our understanding of plant defense evolution. By integrating morphological, evolutionary biological, and molecular biological approaches to systematically explore the multidimensional adaptive mechanisms of thorns, this review provides insights into the evolution of plant defense structures.

## Convergent evolution and coevolution of plant thorn structures

Thorns are morphological innovations that plants have evolved over millions of years to adapt to environmental challenges and have repeatedly evolved independently in different plant groups. Common environmental pressures lead to similar adaptations among organisms. When these adaptations occur independently in unrelated lineages, such traits are considered to have emerged through convergent evolution^[12]^. For example, the wings of birds, bats, and insects are adaptations for flight, but each evolved independently (i.e., convergently) from ancestral wingless species. From the Devonian fossil records to modern plant communities, the emergence and diversification of thorns represent an evolutionary trajectory of long-term interactions between plants and their environment. Spinescent structures of different origins exhibit remarkable convergence in their morphology and function, including leaf thorns, prickles, and stem thorns^[2]^. This convergent evolutionary phenomenon provides important evidence for understanding the driving forces behind the evolution of plants' defensive traits.

Fossil evidence from different geological periods provides a dynamic timeline for the morphological evolution of plant thorns. The earliest known fossil evidence of spinescent structures can be traced back to the Paleogene, approximately 65 million years ago, coinciding with regional aridification and the expansion of open woodland habitats. Over time, the evolution of plant thorns began to exhibit increasingly complex forms. Entering the Eocene epoch (approximately 56 to 34 million years ago), the distribution of spinescent plants expanded significantly, extending from Eurasia into Africa^[13]^. This biogeographic expansion was accompanied by global climate transitions toward increased seasonality and the spread of savanna ecosystems, which likely intensified both abiotic stress and herbivore pressure on vegetation. This process promoted the diversified evolution of thorns' morphology and function, with the advantages conferred by thorns' evolution becoming significantly amplified.

During the late Oligocene epoch, approximately 28 million years ago, large mammals such as *Paraceratheriidae* gradually emerged, followed by the radiation of *Rhinocerotids* in the early Miocene epoch 5 million years later. The population expansion of these large herbivores generated intense feeding pressure on plants and became one of the major driving forces behind the evolution of spinescent defensive structures^[1]^. A significant spatial correlation exists between the distribution of spinescent plants and the activity ranges of large herbivores^[14]^. Furthermore, the emergence of plant thorns forced herbivores to evolve specialized feeding strategies and enhanced digestive capabilities^[5]^. Cacti transformed their leaves into spines, reducing transpirational water loss while effectively deterring herbivore feeding, thereby making consumption difficult for mammals lacking specialized oral adaptations. Camels, however, evolved keratinized lip structures capable of enveloping and processing spinescent leaves to obtain nutritional resources. The rigid thorns of *Acacia* plants can deter most antelopes, but giraffes, relying on their wear-resistant oral mucosa and prehensile tongues, can effectively feed around thorns, occupying a unique ecological niche^[15]^.

**Figure 1:**
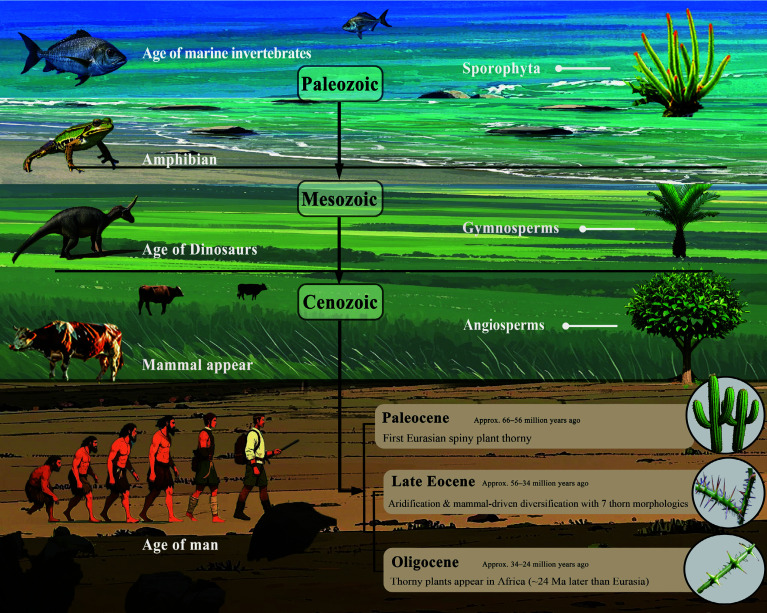
Coevolution of plants and other organisms drives the adaptive evolution of thorns.

From the terrestrial colonization of Devonian spore plants and the emergence of early vertebrates, through the contemporaneous prosperity of Mesozoic gymnosperms and dinosaur dominance, to the rapid expansion of Cenozoic angiosperms and mammalian diversification, the mutual adaptation between plant defenses and animal feeding strategies has driven significant coevolutionary patterns throughout history^[16]^. Meanwhile, environmental factors further modulated the tempo and mode of thorns' evolution. The origin and development of spinescent defensive structures have witnessed the evolutionary arms race between animals and plants spanning tens of millions of years (Fig. 1).

## Tissue origin and structural differentiation of thorns

**Figure 2:**
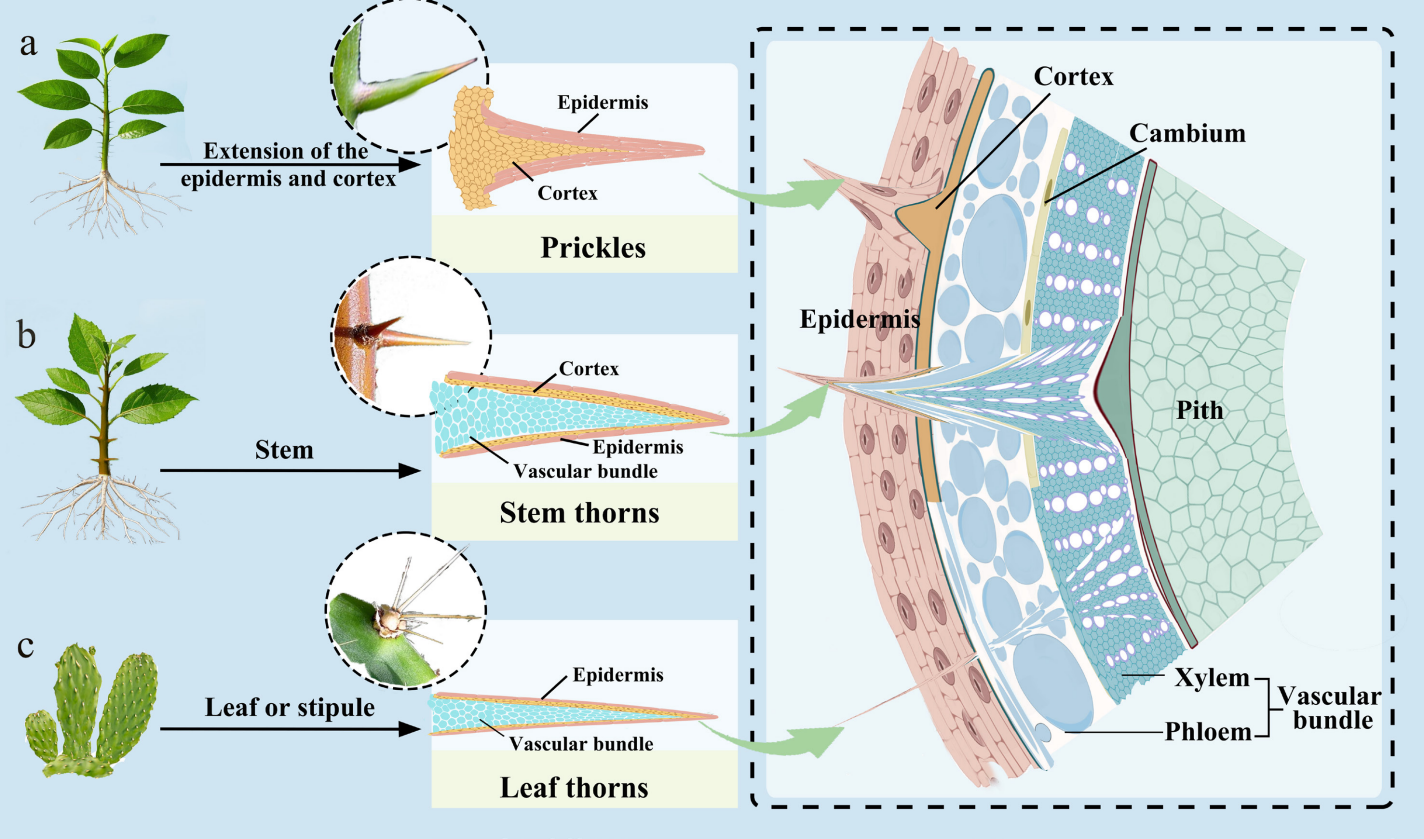
Tissue structure of different types of thorns. (a) Prickles originate from the epidermis and superficial cortical tissues of the stem and are irregularly conical or hooked in shape. They have a thick cuticle on the surface and contain densely packed lignified cells within, lacking vascular tissue. (b) Stem thorns originate from the metamorphosis of branches and have vascular tissue and fully developed xylem within them. (c) Leaf thorns are modified leaves or stipules and usually retain vascular tissue, although their organization differs from that of typical laminar leaves.

Plants face resource allocation constraints when constructing defensive structures, and the balance between functional effectiveness and cost among different types of thorns reflects distinct adaptive strategies shaped by environmental pressures. Depending on the origin site of the thorns on plants, plant thorns can be classified into three main types: Leaf thorns, prickles, and stem thorns^[17]^ (Fig. 2).

Leaf thorns represent an evolutionary outcome whereby plants achieve water conservation and enhanced population adaptability through leaf metamorphosis in extreme water environments. Leaf thorns originate from the metamorphosis of leaves or stipules, with vascular bundles typically showing a discontinuous distribution and often being more prone to mechanical detachment or bending^[18]^. During leaf thorns' development, the direction of cell growth shifts from laminar expansion of the leaf blade to linear growth toward the thorn's apex (Fig. 2c). Cell walls accumulate large amounts of lignin during this process, providing the mechanical strength required for thorns. Notably, leaf thorns may modify the photosynthetically active radiation (PAR) received at the stem's surface, potentially affecting the photosynthetic performance of stems or other photosynthetic organs^[19]^. Surface thorns and marginal thorns (observed in *Ilex cornuta*) are specialized types of leaf thorns formed locally by leaf rachis, leaf veins, or leaf margin tissues^[20]^. Compared with normal leaves, leaf thorns have a small volume and low surface area, with significantly reduced stomatal density, which may allow plants to achieve effective defense at a relatively low developmental cost.

In contrast to leaf thorns, prickles originate from the epidermis and subepidermal tissues of stems and are formed through the rapid proliferation and lignification of locally differentiated cells^[21]^ (Fig. 2a). Structurally, prickles typically present as irregular conical or hooklike formations with thick cuticles on the surface and tightly arranged lignified cells internally. Such anatomical characteristics are likely to influence their functional properties. Because of the lack of vascular integration with the stem, prickles rely only on superficial tissue attachment, making them easily detachable from the stems with smooth fracture surfaces. This detachability may represent an adaptive trade-off: Prickles can provide immediate defense at a low construction cost, and their loss upon herbivore contact does not compromise vascular integrity. Depending on the presence or absence of glandular structures, prickles are classified into nonglandular prickles (NGPs) and glandular prickles (GPs)^[21]^. The former are characterized by uniformly thick-walled cells, which primarily contribute to mechanical deterrence. The latter develop glandular tissues at the apex or base, representing an integration of morphological and chemical defense strategies.

Stem thorns originate from the metamorphosis of shoots, and they containinternal vascular bundle tissues with fully developed xylem, and exhibit high hardness and toughness (Fig. 2b). They are commonly found in plants such as *Crataegus pinnatifida*, *Pyracantha fortuneana*, and *Citrus reticulata*. Stem thorns in different plants vary in their morphology and attachment position. Stem thorns in *Citrus* species mostly arise from axillary meristematic tissues in leaf axils, representing axillary organ metamorphosis. *Pyracantha* stem thorns are commonly attached to axillary short shoots. Stem thorn formation is mechanistically linked to the determination of branch fate. The developmental process involves two types of meristems: Dormant axillary meristems and active thorn meristems. Initially, both appear as domelike protrusions but, depending on environmental and endogenous signals, thorns' primordium cells can gradually elongate into spinescent structures while the axillary meristems may enter dormancy or resume growth, retaining branching capability^[22]^. This developmental flexibility may enable plants to adjust their defensive investment in response to herbivore pressure and resource availability.

Different types of thorns exhibit significant differences in construction cost and defensive effectiveness. Regarding defensive costs, plants may follow the evolutionary logic of "high efficiency and low energy", prioritizing the modification of existing tissues rather than constructing entirely new structures de novo, thereby potentially achieving the dynamic optimization of defensive resources through spatiotemporal differential allocation, and adjusting the density and complexity of defensive structures according to the intensity of environmental threats^[23]^. This strategy is particularly evident in the differentiation of thorn tissues: Prickles generally require only local modification of the outermost cells with a relatively low metabolic cost; leaf thorns can avoid additional vascular system investment by modifying existing leaf tissues, and have been proposed as an early form of thorns' evolution^[24]^; whereas stem thorns, despite requiring higher investment to construct independent vascular and mechanical support systems, may provide longer-term structural stability and defensive persistence. This hierarchical resource allocation pattern ensures plants' adaptability and competitive advantages in complex environments.

## Ecological adaptation strategies of plant thorns

**Figure 3:**
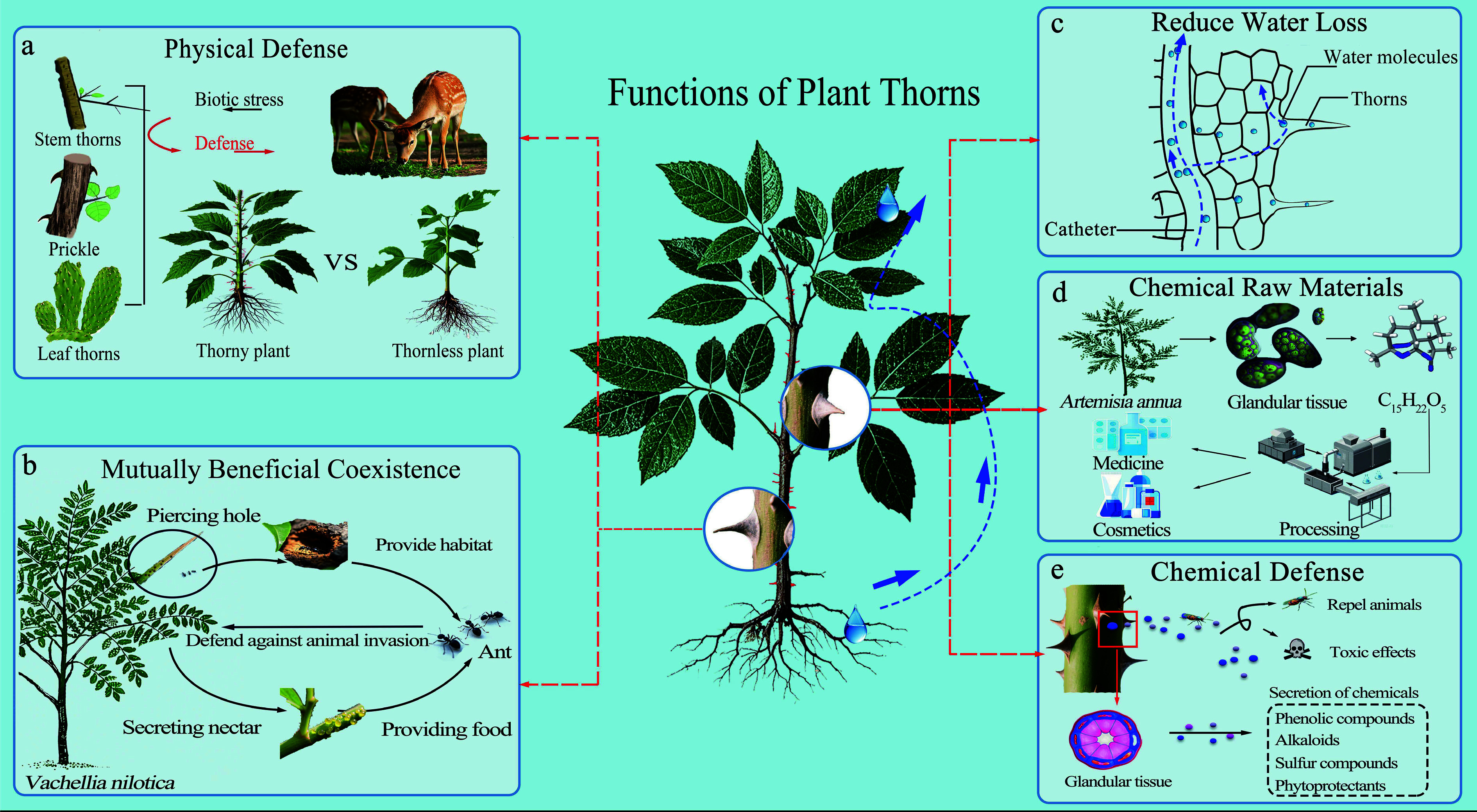
Diverse ecological and economic functions of plant thorns. (a) Physical defense: Various types of thorns serve as both visual deterrents and mechanical barriers, thereby reducing herbivory. (b) Symbiotic interactions: In *Acacia* species, thorns participate in mutualistic associations with ants. The thorns provide nesting sites and are often associated with extrafloral nectaries, whereas the ants protect the host plant from herbivores. (c) Water conservation: By reducing leaf surface area and transpiration, thorns contribute to minimizing water loss, while water continues to be transported upward from the roots. (d) Utilization of metabolites: Secondary metabolites stored in glands associated with thorns can be exploited for the production of spices, essential oils, pharmaceuticals, and food additives. (e) Chemical defense: Glandular thorns synthesize and accumulate diverse secondary metabolites, which have repellent or toxic effects to herbivores.

According to Givnish's adaptive radiation theory^[25]^, the evolution of multifunctional organs enables plants to achieve functional optimization in environments with limited resources, with the diversified functions of thorns representing a manifestation of this evolutionary strategy. Over the course of evolution, plant thorns have developed into multifunctional integrated systems rather than purely defensive organs. This functional diversification stems from the complex environmental challenges plants face. Compared with thornless plants, thorny plants possess greater advantages in terms of resource competition and survival under harsh environments. From simple mechanical protection to complex biochemical regulation, and from individual adaptation to interspecific mutualism, thorns demonstrate the high integration of plants' adaptive strategies (Fig. 3).

Plant thorns construct multilayered physical defense systems. Before contact, herbivores significantly increase their vigilance time and reduce their feeding residence time when facing thorny plants^[26]^. The visual warning and structural deterrence of thorns compel herbivores to modify their behavioral patterns, thereby reducing actual physical contact and damage. At the mechanical level, the physical barrier effect of thorns provides direct protection. Rosaceae plants such as *Rosa chinensis*, *Rosa roxburghii*, and *Pyracantha fortuneana* commonly develop hard thorns on their branches and leaf margins, exemplifying this defensive strategy (Fig. 3a). Environmental plasticity further optimizes defensive efficiency, with plants under high feeding pressure increasing their thorns' length and density. This induced adaptive response enables plants to adjust defensive resource allocation according to actual feeding pressure^[27]^.

The co-evolution of herbivores and plants has created unique mutualistic niches associated with plant thorns. In some *Vachellia farnesiana* species, thorns are modified into enlarged hollow domatia that provide shelter for ants and food resources through basal nectaries. Ants, in turn, help acacias resist herbivorous animals through defensive behavior and selectively removing the seedlings of other plants competing with acacias. This exclusive protective mechanism enables ant-inhabited acacias to gain significant competitive advantages in plant communities, with enhanced growth rates and survival rates significantly higher than those of individuals lacking ant protection^[28]^ (Fig. 3b). Similarly, the dense thorn structures of *Carnegiea gigantea* provide a habitat space for different animal groups along a vertical gradient. Thorn clusters at the ground level provide a shelter and refuge for small rodents, whereas the upper thorn clusters serve as nesting and foraging habitats for birds. Animals reciprocate to the host plants through predation, nesting, defecation, and other behaviors, forming mutually beneficial and stable ecological network relationships^[29]^. These interspecific interactions mediated by thorns enhance the community's stability and contribute to ecosystem diversity.

Furthermore, thorns help plants reduce transpiration by regulating local moisture environments and provide additional water sources by collecting moisture. In arid environments, leaf specialization into thorn structures significantly reduces transpiration area. The Euphorbiaceae species *Euphorbia milii* has evolved leaves into needlelike structures, significantly reducing water loss rates (Fig. 3c). Dense thorn structures form relatively static air layers around plant bodies, reducing the effects of wind speed on plant surfaces and increasing local humidity. Thorns may also facilitate moisture capture, as the surface tension effects of dense thorn structures can capture atmospheric moisture, thereby providing additional water sources for plants under morning dew conditions. Additionally, dense thorns can create small-scale shading conditions that reduce direct solar radiation, lower plant surface temperatures, and alleviate the intensity of temperature stress^[7]^.

Beyond mechanical defense, certain sharp appendages function via a combination of physical and chemical mechanisms, forming an integrated defense strategy. Recent studies suggest that prickles are often derived from modified glandular trichomes and can accumulate secondary metabolites. Upon herbivore contact, these structures can release bioactive compounds through secretory tissues, causing irritation or deterrence^[30]^. Similarly, glandular trichomes store high concentrations of metabolites in subcuticular cavities between the glandular cell walls and cuticles^[31]^, enabling rapid responses in passive defense. For example, the terpenoids that are enriched in *Artemisia annua* support a wide range of applications, including pharmaceuticals and other commercial products^[32]^ (Fig. 3d). Additionally, polysaccharide substances resist mechanical damage by enhancing thorns' cell wall strength and participate in plants' immune regulation. Organic acids serve as chemical signals to repel herbivores while inhibiting pathogenic microorganisms' growth. Phenolic compounds and terpene volatiles in *Rosa* species thorns possess antibacterial and insecticidal properties. Alkaloids accumulated in cactus leaf thorns can absorb ultraviolet radiation and induce reactive oxygen species generation, creating microenvironments that are unfavorable for the survival of parasites and pathogens^[33]^. The biological activity of metabolites in thorns makes them defensive structures with multiple functions, including antibacterial, insecticidal, and antioxidant actions^[34]^ (Fig. 3e). The same individual plant may possess multiple types of thorns simultaneously, corresponding to different ecological functions. This spatially heterogeneous defense deployment enables plants to obtain more comprehensive protection when facing herbivores with different feeding preferences.

## Molecular regulatory mechanisms of thorns' development

### Transcriptional regulatory networks controlling thorns' development

The phenotypic diversity and environmental responsiveness of thorns are subject to sophisticated molecular regulation. Although the molecular mechanisms underlying thorns' development remain incompletely elucidated, multiple studies have revealed intrinsic connections between thorns and trichomes at the molecular regulatory level^[17,35,36]^. Early morphological observations proposed that prickles may represent modified epidermal structures related to glandular trichomes that undergo lignification during development. Although prickles in woody plants differ morphologically from trichomes in herbaceous plants, both originate from the directed differentiation of epidermal cells and may contribute to plants' defense and environmental adaptation^[37−39]^. The molecular regulatory framework established through trichome research provides a comparative framework for understanding the molecular basis of prickle development, and may offer insights into other thorn types (Table 1).

**Table 1:** Reported regulators and candidate genes associated with thorns' development, together with comparative evidence from trichome systems.

Species	Thorn type	Key regulator	Regulatory role	Experimental evidence	Regulatory relationship	Ref.
*Rosa rugosa*	Prickles	*RrCPC*	Negative regulatory factor	Functional validation (transgenic in *Arabidopsis*)	*RrCPC* and MBW complex exhibit significant competitive inhibitory effects	[52]
*Rosa roxburghii*	Prickles	*RrTTG1*	Positive regulatory factor	Functional validation (transgenic in *Arabidopsis*)	Interaction between *RrTTG1* and *RrEGL3*	[53]
*Oryza sativa* L.	Trichomes	*OsSPL10*	Positive regulatory factor	Functional validation (gene editing, knockout, hybrid analysis)	*OsSPL10* positively regulates trichome development through auxin signaling pathways	[54]
*Arabidopsis thaliana*	Trichomes	*GLABRA2*	Positive regulatory factor	Functional validation (transgenic analysis)	*GL2* determines trichome cell fate downstream of the MBW complex	[55]
		*TRICHOMELESS2*	Negative regulatory factor	Functional validation (overexpression and knockout in *Arabidopsis*)	*TCL2* competes with *GL1* for binding to *GL3*/*EGL3*, inhibiting trichome initiation	[45]
		*GCN5*	Positive regulatory factor	Functional validation (overexpression and knockout in *Arabidopsis*)	*GCN5* promotes trichome initiation through histone acetylation	[56]
		*GLABROUS INFLORESCENCE STEMS* (*GIS*)	Positive regulatory factor	Functional validation (overexpression and knockout in *Arabidopsis*)	*GIS* promotes trichome initiation and enhances *GL1* expression in transgenic *Arabidopsis thaliana*	[57]
		*TTG1*	Positive regulatory factor	Genetic evidence (genetic interaction analysis)	*TTG1*, *GL3*, and *GL1* form complexes *in vivo* to positively regulate the formation of trichomes	[58]
		*CPC*	Negative regulatory factor	Functional validation (transgenic analysis)	*CPC* negatively regulates the expression of *GL2*, and transgenic plants overexpressing the *CPC* gene exhibit the same phenotype as *GL2* mutants	[59]
		*TRIPTYCHON*	Negative regulatory factor	Functional validation (transgenic analysis)	Inhibits trichome initiation through competitive inhibition within the MBW complex	[60]
		*GLASSY HAIR* (*GLH*)	Positive regulatory factor	Functional validation (transgenic analysis)	Involved in the metabolism or deposition of trichome cell wall components	[61]
*Citrus reticulata*	Stem thorns	*THORN IDENTITY1*, *THORN IDENTITY2*	Positive regulatory factor	Functional validation (transgenic overexpression in citrus)	Promotes the conversion of axillary branches into thorns	[22]
		*CENTRORADIALIS*	Negative regulatory factor	Functional validation (transgenic overexpression in *Citrus*)	*CsCEN* antagonizes *TI1*/*TI2* and regulates the transition between thorn and branch identity	[62]
*Solanum lycopersicum*	Trichomes	*SlCD2*	Positive regulatory factor	Genetic evidence (mutant phenotype analysis)	*CD2* is a major regulator of tomato epidermal cell function	[63]
		*SlMIXTA*	Positive regulatory factor	Functional validation (overexpression)	Primary metabolism can be reprogrammed to produce specialized metabolites and form trichomes as storage compartments	[64]
		*SlZFP8*, *SlZFP6*	Positive regulatory factor	Functional validation (overexpression and knockout)	*SlZFP8* is involved in the control of trichome elongation and *SlZFP6* is a downstream target for the control of trichome elongation	[65]
		*SlARF3*	Positive regulatory factor	Genetic evidence (knockout)	*SlARF3* plays an important role in epidermal cell formation and differentiation	[66]
*Zanthoxylum bungeanum*	Prickles	*ZaHDZ16*	Positive regulatory factor	Correlative evidence (gene structure and motif analysis)	*ZaHDZ16* is associated with thorns' development on branches	[67]
		*ZaMYB86*	Negative regulatory factor	Functional validation (transgenic in *Arabidopsis*)	*ZaMYB86* may regulate prickle development through hormone signaling pathways	[68]

Direct molecular evidence for thorn development remains limited, and research findings on model organisms such as *Arabidopsis thaliana* serve as a reference for understanding epidermal cell differentiation. In *Arabidopsis*, *GLABRA 1* (*GL1*) is the first gene identified to be associated with trichome development. Its MYB domain interacts with the basic helix–loop–helix (bHLH) transcription factors *GLABRA 3* or *ENHANCER OF* *GLABRA 3* (*GL3*/*EGL3*), regulating the differentiation fate of plants' epidermal cells and directly affecting trichome formation^[40−42]^. The regulatory network centered on the MYB–bHLH–WD40 (MBW) complex, comprising *GL1*-*GL3*/*EGL3*-*TTG1*, occupies a central position in trichome development. This complex binds to *cis*-acting elements within the promoter region of the downstream gene *GL2* and activates its transcription, thereby determining epidermal cells' fate toward trichome differentiation^[43]^. Concurrently, the MBW complex achieves precise regulation through a negative feedback mechanism: While activating *GL2*, it also induces the expression of genes encoding single-repeat R3-MYB proteins, such as *CPC*^[44]^, *TRICHOMELESS2*^[45]^, and *ETC1*^[45,46]^. This regulatory framework provides important clues for a deeper understanding of how transcriptional networks regulate epidermal specialization. However, it is important to emphasize that the current evidence does not indicate that this pathway directly controls the formation of leaf thorns, stem thorns, or prickles. The trichome regulatory system should be considered more as a comparative framework to help guide the identification of candidate genes involved in the development of sharp plant appendages.

Studies have demonstrated that this regulatory network exhibits a degree of functional conservation across multiple plant species. Homologs of several key regulatory genes have been shown to be involved in the thorn fate differentiation of epidermal cells in various plants, including roses and camellias^[47]^. In *Rosa roxburghii*, *RrGL1* shows high sequence conservation with *Arabidopsis* *GL1*, and its encoded protein interacts with GL3/EGL3, suggesting its possible involvement in the early morphogenesis of prickles^[21]^. In *Zanthoxylum bungeanum*, candidate genes including *ZbYABBY2*, *ZbYABBY1*, *ZbYABBY5*, *ZbWRKY*, *ZbLoG5*, *ZbAZG2*, *ZbGh16*, *ZbIAA33*, and *ZbGh16X1* show elevated expression during prickle development^[48,49]^. Although these studies rely primarily on gene expression correlations and functional validation remains limited, they suggest that certain components of the epidermal regulatory network may be involved in the initiation or differentiation of sharp plant appendages.

Moreover, thorns originate from different tissues and organs, which implies that their regulatory pathways may vary substantially. Prickles arise primarily from epidermal or cortical tissues, whereas stem thorns originate from modified shoots, and leaf thorns originate from modified leaves. Different types of thorns may involve partially independent genetic regulatory systems. For instance, in *Citrus*, stem thorns originate from axillary bud development, depending on the synergistic action between TCP transcription factors (*TI1*/*TI2*) and *CsCEN*, which switch between thorn and branch fates by regulating meristems' quiescence and activation states^[22]^. Recent work has further identified *TI3*, a member of the *SHI*/*STY* transcription factor family, as an upstream regulator in this process. *TI3* directly binds to a CTAG core element in the promoters of *TI1* and *TI2*, thereby activating their expression and promoting stem cell arrest in the developing thorn meristem. In contrast, *CsCEN* represses *TI3* expression in axillary meristems, maintaining meristem activity and enabling branch outgrowth^[50]^. The lignification process of *Rosa multiflora* prickles is mainly regulated by the NAC transcription factor *RmNAC43*, which promotes secondary wall thickening and lignification of prickles by upregulating lignin biosynthesis genes^[51]^.

Overall, the available evidence suggests that the development of sharp plant appendages may involve a partially conserved regulatory logic together with substantial lineage specific divergence. Specifically, the well-characterized trichome regulatory networks provide a foundational model for understanding the development of epidermally derived prickles. Meanwhile, different plant lineages appear to have evolved additional lineage-specific developmental pathways and regulatory factors, resulting in the morphological and functional diversification of sharp appendages. This hypothesis highlights a possible balance between the conservation of key regulatory modules and lineage-specific innovation, a premise that requires further experimental validation.

Furthermore, research on plant trichomes has gradually extended into the field of epigenetics. Epigenetic regulatory factors control gene expression through multiple pathways, participating in decisions on thorns' developmental fate as "cryptic regulatory mechanisms" in epidermal specialized structure formation^[69,70]^. DNA methylation, histone modifications, and noncoding RNAs regulate gene expression without altering the DNA sequence, providing an additional layer of regulation for the development of plants' epidermal structures. Current research mainly focuses on post-transcriptional RNA modifications and regulation mediated by microRNA (miRNA), whereas the understanding of mechanisms such as chromatin remodeling and protein homeostasis remains relatively limited^[71]^.

DNA methylation regulates methylation patterns in the promoter regions of key developmental genes, affecting transcription factor binding and gene expression levels. In tomato (*Solanum lycopersicum*), demethylase *SlIDM3* forms functional complexes with *HAP2*/*3*/*5* transcription factors, targeting the demethylation of genes specific to trichomes and affecting their alternative splicing patterns. The *slidm3* loss-of-function mutants exhibit phenotypes with significantly increased trichome density^[72]^. Systematic analysis of multiple DNA methyltransferase mutants in *Arabidopsis* (including *met1*, *drm1*, *drm2*, *cmt3*, and *cmt2*) also reveals that differential methylation modifications at CG and non-CG sites have irreplaceable regulatory functions for normal trichome development programs^[73]^. Evidence for thorns' development in woody plants is more direct. Comparative analysis of transcriptomes and methylomes between thorny and thornless varieties of *Zanthoxylum armatum* shows a significant positive correlation between hypomethylation states in the promoter regions of genes related to thorn development and their high expression levels. DNA methyltransferase genes are downregulated in thorny individuals, suggesting that reduced methylation levels may promote the activation and expression of thorn development genes^[70]^.

Histone modifications establish specific chromatin epigenetic markers, precisely regulating the activation states of transcriptional regulatory networks of thorn development. Histone deacetylase *DEACETYLASE 6* (*HDA6*) and acetyltransferase *GCN5* dynamically regulate acetylation levels in the promoter regions of key trichome genes, including *GL1*, *GL2*, *GL3*, and *CPC*, thereby influencing trichome initiation and offering comparative insights into epidermally derived prickles^[56]^. Ubiquitin-protein ligase 3 (*UPL3*) specifically degrades *GL3* and *EGL3* transcriptional activator proteins through modifying ubiquitination, negatively regulating trichome branching development at the protein level^[74]^.

Noncoding RNAs are key participants in epigenetic modifications, including long noncoding RNAs (lncRNAs), small interfering RNAs (siRNAs), and microRNAs (miRNAs). MicroRNAs (miRNAs) are endogenous noncoding small RNAs that are widely present in plants that achieve the fine regulation of gene expression through base pairing with the target mRNAs, causing their degradation or translational inhibition^[71]^. In *Arabidopsis*, *miR156* targets the degradation of *SQUAMOSA PROMOTER BINDING PROTEIN-LIKE* (*SPL*) family member mRNAs. Specifically, the SPL9 protein directly binds to the promoters of the trichome inhibition genes *TCL1* and *TRY*, negatively regulating trichome development independently of the *GL1* pathway^[75]^. *miR319* indirectly activates the transcription of the trichome branching inhibitor *GIS* by suppressing *TCP4* expression, thereby regulating trichome branching patterns and final morphogenesis^[76]^. Because of their reversibility and environmental responsiveness, epigenetic mechanisms are considered to be a critical bridge linking genotype to phenotypic plasticity. As thorns represent an important structural adaptation in plant evolution, epigenetic regulation is expected to open new avenues for understanding the molecular basis of thorns' phenotypic diversity.

### Hormones as signals mediating the regulatory network of thorn formation

Phytohormones play important roles in regulating epidermal differentiation, organ development, and defensive trait formation^[77]^. Teh current knowledge mainly derives from studies in model plants, where several hormones such as gibberellic acid (GA), jasmonic acid (JA)^[78,79]^, cytokinin (CK), salicylic acid (SA), brassinosteroids (BRs)^[80]^, and ethylene (ET) have been shown to influence epidermal cells' differentiation and the formation of specialized structures^[81]^. These studies provide a conceptual framework for understanding how hormonal signals may influence the differentiation of epidermal appendages.

In contrast, several studies have provided more direct evidence linking hormone signaling pathways to the development of plant thorns in specific species. The *LONELY GUY* (*LOG*) family encodes cytokinin nucleoside 5'-monophosphate phosphohydrogenases, catalyzing the final step in cytokinin activation. Cytokinin activation mediated by *LOG* is thought to directly influence the development of epidermal structures, including thorn formation. In eggplant (*Solanum melongena*), the *PRICKLELESS* (*PL*) gene encodes a LOG protein, and the *pl* mutant exhibits a phenotype without prickles^[82]^. The *RcLOG1* gene in *Rosa* is also closely related to prickle development^[83]^, further supporting the role of cytokinin in regulating the initiation of epidermal appendages in plants. Recent work in *Citrus* has provided new insights into the genetic regulation of thorn formation. A member of the *SHI*/*STY* transcription factor family, *TI3*, was shown to bind CTAG motifs in the promoters of multiple genes, including the auxin biosynthetic genes *CsYUC2* and *CsYUC8*, suggesting a potential role in coordinating thorn development through auxin-mediated pathways. Interestingly, thorn production in *Citrus* is strongly associated with the juvenile phase and can reappear in shoots induced by stress, such as epicormic sprouts. Thorn identity genes may be regulated by both developmental programs and environmental signals. These findings suggest that the *SHI*/*STY*–auxin module may represent an important regulatory component in thorns' development, although the broader regulatory network remains largely unresolved^[50]^.

Hormonal signaling also promotes the development and maturation of sharp plant appendages by participating in cell differentiation and lignification. The gibberellin signaling pathway is closely related to cell wall thickening and lignification^[84]^. Members of the DELLA protein family, namely *GAI*, *RGA*, and *RGL*, promote cell wall thickening and lignification by inhibiting GA signaling^[57]^, a process that is crucial for the mechanical stiffness of defensive structures such as thorns. Similarly, ET signaling plays a major role in trichomes' maturation and lignification, participating in cell wall lignification through signal receptors such as ETR2 and EIN2, which may contribute to the maturation of defensive structures^[85]^.

**Figure 4:**
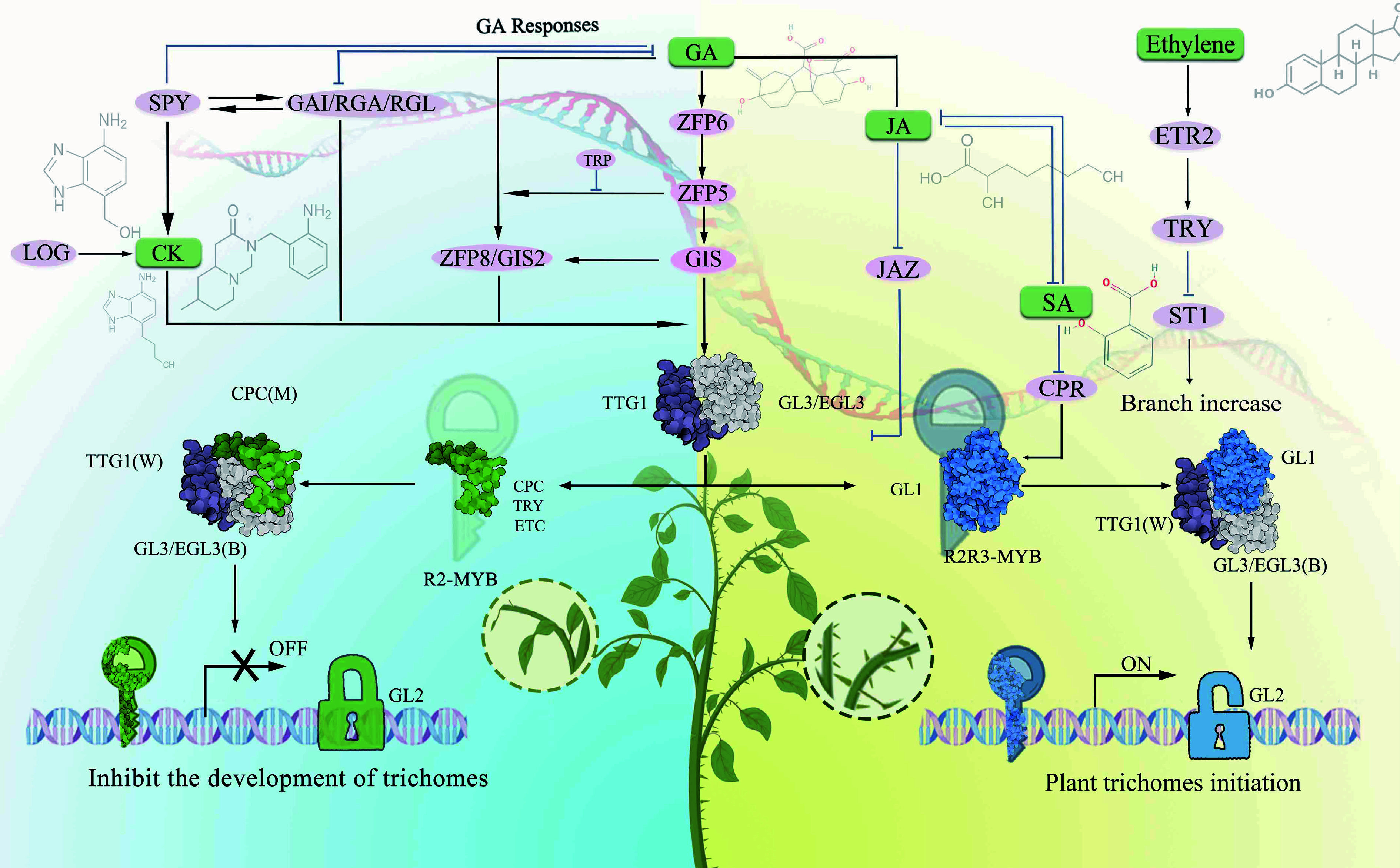
Molecular regulatory model of plant trichomes' differentiation and development.

Furthermore, hormones function as signaling molecules, and exhibit synergistic and antagonistic interactions during epidermal structures' morphogenesis. In trichome development, GA and JA demonstrate synergistic effects, with exogenous GA application significantly enhancing the promoting effect of JA on trichome density. On one hand, JA suppresses the expression of GA biosynthesis genes through the GA–DELLA pathway, reducing local GA concentrations and thereby indirectly modulating trichome development^[86]^. On the other hand, JA affects the *ARR* transcription factor's activity in cytokinin signal transduction, regulating epidermal cells' division and differentiation^[87]^. CK and BRs jointly participate in morphogenesis processes during the trichome elongation stage, regulating the final size and mechanical strength of trichomes. The antagonistic and synergistic relationships among hormones provide plants with flexible regulatory mechanisms, enabling dynamic adjustment of defensive structures' developmental strategies in changing environments (Fig. 4).

## Discussion

Current research on plants' spinescent structures has improved our understanding of their developmental diversity, ecological functions, and regulatory basis, but major mechanistic gaps remain. By integrating paleobotanical evidence, anatomy, and modern molecular biology studies, this review suggests that thorns have evolved independently as products of convergent evolution across different phylogenetic lineages, and highlights their spatiotemporal associations with the coevolution of large herbivorous mammals. At the molecular regulatory level, certain epidermally derived thorns share profound intrinsic connections with trichomes. According to the known molecular regulatory mechanisms of trichome development, particularly the regulatory network centered on MBW, and in combination with epigenetic modifications and hormone signaling, this framework provides an important theoretical reference for investigating the molecular mechanisms of thorns' development.

Current molecular research on thorns has primarily remained at the level of homologous gene identification and expression correlation analyses, and the complete molecular regulatory network governing thorns' development has yet to be systematically elucidated. Compared with the regulatory mechanisms of unicellular trichomes, the number of genes investigated in thorn-related developmental regulatory models remains limited, and the similarities and specificities of regulatory mechanisms among different plant lineages require further clarification. Based on existing gene networks, the integration of transcriptomics and functional genomics provides opportunities for identifying potential regulatory genes in thorns' development^[88]^. Recent studies have revealed that the lignification of thorn tips is a key determinant of their defensive function. In *Citrus*, the MYB transcription factor *SHORT* and *SOFT THORN 1* (*SST1*) acts as a central regulator of thorn tip hardening. *SST1* is directly activated by thorn identity genes *TI1*/*TI2* and subsequently induces a hierarchical transcriptional network involving NAC transcription factors and their downstream regulators, thereby promoting secondary cell wall biosynthesis and sclerenchyma differentiation in thorn tips^[89]^. Notably, the highly specific spatiotemporal expression of *SST1* provides a potential strategy for breeding varieties with soft thorns without affecting overall plant development.

The evolutionary interactions between plants and animals manifest as an ongoing process of selective pressures and adaptive responses. Plants limit herbivory through morphological specialization and metabolic defense mechanisms in thorns, whereas animals continually evolve new foraging strategies to break through the plants' defenses. The interaction between plants and animals is fundamental to the structure and function of ecosystems^[90]^. Rather than viewing this as a simple confrontation, it can be understood as a dynamic adaptive balance. This balance not only shapes individual traits but profoundly impacts populations' size and stability^[91]^. At the community level, plants' defense mechanisms indirectly determine the nutritional resources and reproductive rates of herbivores, whereas the selective pressures exerted by animals drive the maintenance of plant diversity and the emergence of new traits, resulting in a dynamic balance in species richness and functional diversity within the community^[92]^. Excessive or insufficient selective pressure may disrupt this balance, threatening the health of the community. The interaction between plants and animals promotes ecological functions by maintaining biodiversity, influencing the plant population's dynamics, and providing key ecosystem services. However, the biological mechanisms underlying these interspecies interactions remain poorly understood. In the future, the advent of machine learning and artificial intelligence offers powerful new methods for analyzing complex, multidimensional datasets generated from studies over the long term. Advances in analytical techniques, combined with more powerful computing capabilities, may uncover subtle patterns and relationships that traditional statistical methods could overlook^[93]^. As an important mechanical defense mechanism, thorn traits provide an ideal research entry point for understanding the interactions between plants and animals and their sensitivity to environmental changes.

## Conclusions

Thorns are adaptive structures resulting from long-term plant adaptation to herbivore pressure and environmental stress. Diverse thorns represent differentiated adaptive strategies balancing defense durability, construction cost, and environmental responsiveness. This structural diversity is not stochastic but reflects an evolutionary strategy of optimizing resource allocation across different environments. Current research on the regulatory networks of trichome initiation has laid the foundation for further exploration of potential regulatory factors in thorns' development across different species. Through the integration of transcriptional, metabolic, and epigenetic regulatory interactions, combined with comparative studies across species and functional validation studies, a more comprehensive understanding of the regulatory processes of thorn initiation is expected. In the context of global environmental change, integrating molecular regulation with environmental information will help establish links between thorns' developmental regulation and environmental adaptation. Meanwhile, with the advancement of research on spinescent species, understanding the developmental mechanisms of thorns will not only facilitate the breeding of thornless varieties that are easier to harvest while maintaining other desirable traits, but may open new perspectives for a comprehensive understanding of plants' environmental adaptability and evolutionary potential.

## Data Availability

Data sharing is not applicable to this article, as no datasets were generated or analyzed during the current study.
